# FEM Simulation of a High-Performance 128°Y–X LiNbO_3_/SiO_2_/Si Functional Substrate for Surface Acoustic Wave Gyroscopes

**DOI:** 10.3390/mi13020202

**Published:** 2022-01-27

**Authors:** Rui Ma, Weiguo Liu, Xueping Sun, Shun Zhou, Dabin Lin

**Affiliations:** 1 School of Microelectronics, Xidian University, Xi’an 710071, China; marui@xatu.edu.cn; 2 Shaanxi Province Key Laboratory of Thin Films Technology and Optical Test, School of Optoelectronic Engineering, Xi’an Technological University, Xi’an 710032, China; sunxueping.1988@163.com (X.S.); zsemail@126.com (S.Z.); dabinlin@xatu.edu.cn (D.L.)

**Keywords:** surface acoustic wave gyroscope, 128°Y–X LiNbO_3_/SiO_2_/Si substrate, Rayleigh wave, gyroscopic effect, finite element simulation

## Abstract

To obtain a high-performance surface acoustic wave (SAW) gyroscope substrate, the propagation characteristics and gyroscopic effect of Rayleigh waves in a 128°Y–X LiNbO_3_/SiO_2_/Si (LNOI) functional substrate were investigated with a three-dimensional finite element method. The influence of LNOI structural parameters on Rayleigh wave characteristics, including the phase velocity (v_p_), electromechanical coupling coefficient (K^2^) and temperature coefficient of frequency (TCF), were analyzed. The results demonstrate that the SiO_2_ layer compensates for the negative TCF of 128°Y–X LiNbO_3_ and enhances the K^2^ of the LNOI substrate. The Rayleigh wave velocity change of the LNOI substrate after rotations in different directions was studied. The gyroscope gain factor (η) represents the strength of the gyroscopic effect in the differential traveling wave SAW gyroscope and is defined. The η_y_ and η_z_ of the LNOI substrate with different structural parameters were investigated. Finally, an LNOI substrate with an h_LN_/λ of 0.2 and an h_SiO2_/λ of 0.05 was obtained by balancing the characteristic parameters, with a K^2^ of 3.96%, TCF of −18.75 ppm/°C and η_y_ of 0.26. The LNOI substrate has a better gyroscopic effect and temperature stability than the 128°Y–X LiNbO_3_ crystal. The LNOI substrate meets device miniaturization and integration needs.

## 1. Introduction

The surface acoustic wave (SAW) gyroscope is a novel inertial sensor based on the SAW gyroscopic effect in which the Coriolis force causes the SAW velocity to change as the medium rotates [1]. The SAW gyroscope measures the angular velocity by detecting the frequency shift caused by a change in the wave velocity [2]. Compared with traditional mechanical rotor gyroscopes and vibratory gyroscopes, the SAW gyroscope is a single-layer planar structure with no moving parts and an elastic support structure with strong shock resistance and vibration resistance. In addition, SAW gyroscopes have the same advantages as SAW devices, such as small size, digital output, high sensitivity, and simple manufacturing [3,4,5]. The SAW gyroscopes that have been reported to date contain mainly standing wave and traveling wave modes. Research on standing wave SAW gyroscopes has been paused due to their too weak output signal, complex device structure and temperature compensation difficulty [6,7,8]. Traveling wave SAW gyroscopes have attracted widespread attention because their output signal is easy to detect and temperature compensation can be achieved with a differential scheme [9,10]. However, SAW gyroscopes are difficult to use in practical applications because of their low sensitivity due to the weak Coriolis force. Improving the gyroscopic effect to obtain a high-sensitivity SAW gyroscope is still an urgent problem that must be solved to overcome current application limitations. Many research groups have conducted intensive studies on designing interdigital transducers, placing metallic dot arrays on the surface of piezoelectric crystals, modulating amplitude using one-dimensional phononic crystal, and searching for high-performance piezoelectric substrate materials to further improve the performance of SAW gyroscopes [11,12,13,14,15].

High-performance substrate materials are an important foundation for manufacturing high-sensitivity SAW gyroscopes. A high-performance substrate material for an SAW gyroscope must have a high sensitivity to gyroscopic effects, a large electromechanical coupling coefficient (K^2^) and a temperature coefficient of frequency (TCF) that approaches zero. For different piezoelectric crystals, the strength of the gyroscopic effect is affected by their physical characteristics. For the same piezoelectric crystal, the SAW gyroscopic effect of different cut types differs because of their anisotropic characteristics [12,16]. Currently, most SAW gyroscopes are prepared on piezoelectric crystal wafers with clear gyroscopic effects, such as ST-quartz, 128°Y–X LiNbO_3_ and X–112°Y LiTaO_3_ [9,10,17,18]. A traveling wave SAW gyroscope achieves phase velocity changes by measuring the frequency shift caused by loading a rotation, so the substrate must have good temperature stability. Although ST-quartz with a TCF of zero can meet the above requirement, its K^2^ is only 0.14%, which has a considerable effect on the bandwidth of the device. X–112°Y LiTaO_3_ has a TCF of −20 ppm/°C and K^2^ is also of only 0.75%. The K^2^ of 128°Y–X LiNbO_3_ is 5.5%, but its TCF is −75 ppm/°C. To eliminate the temperature effect, a double delay line differential structure was designed on the 128°Y–X LiNbO_3_ wafer. The inherent characteristic parameters of the substrates mentioned above limit improvements in the SAW gyroscope performance. Recently, a piezoelectric multilayer structure that combines a piezoelectric film and a silicon substrate has attracted widespread attention. The angular velocity can be measured by using generalized Rayleigh waves in the layered structure [19]. The piezoelectric multilayer structure can be composed of materials with different characteristics and meets the requirements of SAW devices through its structural design, which is very flexible [20,21,22]. Additionally, this structure can efficiently reduce the volume and weight of the SAW sensor.

**Figure 1:**
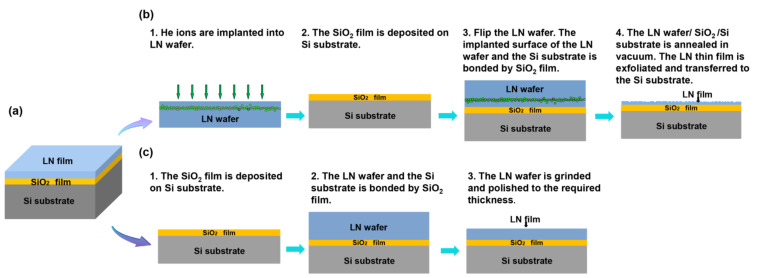
(**a**) Schematic diagram of the LNOI substrate structure; (**b**) preparation flow chart of the LNOI substrate with a nanoscale LN film; (**c**) preparation flow chart of the LNOI substrate with a micron-order LN film.

Among piezoelectric multilayer structures, a lithium niobate thin film on insulator (LNOI) substrate has been used in the preparation of SAW sensors due to its excellent piezoelectric properties [23,24,25,26]. As shown in Figure 1a, this substrate is composed of a LiNbO_3_ (LN) thin film, a SiO_2_ layer and a silicon (Si) substrate. In this structure, the Si substrate at the bottom is mainly used for mechanical support and subsequent circuit integration. The intermediate layer SiO_2_ is a common waveguide material with low noise loss and high mechanical and chemical properties [27]. The SiO_2_ layer not only acts as a bonding layer between the LN thin film and the Si substrate but also plays an important role in adjusting the TCF of the LNOI substrate because of its positive TCF. As the piezoelectric layer, the LN thin film at the top of the structure is the core of the LNOI substrate. The cut type, film quality and thickness of the LN thin film directly affect the performance of the LNOI substrate. Currently, there are two mature LNOI substrate preparation processes according to the thickness of the LN thin film [28]. As shown in Figure 1b, smart-cut technology is applied to prepare the LNOI substrate with a nanoscale LN film. First, He ions are injected into the LN wafer, and then a SiO_2_ thin film is deposited on the silicon substrate using plasma-enhanced chemical vapor deposition (PECVD). The implanted LN wafer is flipped and bonded to a silicon wafer by the SiO_2_ layer. After the annealing step, an LN thin film is split off from the original LN wafer, yielding an LNOI substrate. Finally, the surface roughness of the LN film can be polished to 0.5 nm by chemical mechanical polishing (CMP) [29,30]. An LNOI substrate with a special cut type and a high single-crystallinity LN thin film can be prepared by the above method. However, the thickness of the stripped LN film is determined by the He ion implantation energy, so this method is not suitable for preparing LN thin films thicker than a few microns. The preparation scheme for the LNOI substrate with an LN film thicker than a few microns is shown in Figure 1c. First, the LN wafer is bonded to the Si substrate through a SiO_2_ layer. Then, the LN wafer is removed by grinding to obtain the LN thin film. Finally, the surface of the LN film is polished by CMP. The mature preparation process establishes the groundwork for the widespread use of LNOI substrates. According to the literature, the optimized X-cut LNOI substrate has a K^2^ value of up to 35% and a TCF value of −0.2 ppm/°C for shear-horizontal (SH) waves [31]. It is a high-performance substrate for preparing SH-SAW and Lamb wave devices with a wide band and low loss [32,33]. This LNOI substrate can achieve a larger K^2^ and lower TCF by optimizing the structural parameters, providing a new possibility for obtaining a high-performance substrate for SAW gyroscopes. To date, there has been no discussion of the SAW gyroscopic effect in LNOI substrates.

In this paper, a three-dimensional finite element model was established to analyze the SAW propagation characteristics of a 128°Y–X LiNbO_3_/SiO_2_/Si substrate (in this paper, LN represents 128°Y–X LiNbO_3_). The influence of LNOI structural parameters on the phase velocity (v_p_), K^2^, TCF and gyroscopic gain factor (η) were investigated in detail. The thicknesses of various layers were optimized to obtain an LNOI substrate with a high K^2^ and η and a low TCF.

## 2. Gyroscopic Effect and Simulation Details

### 2.1. Gyroscopic Effect

**Figure 2:**
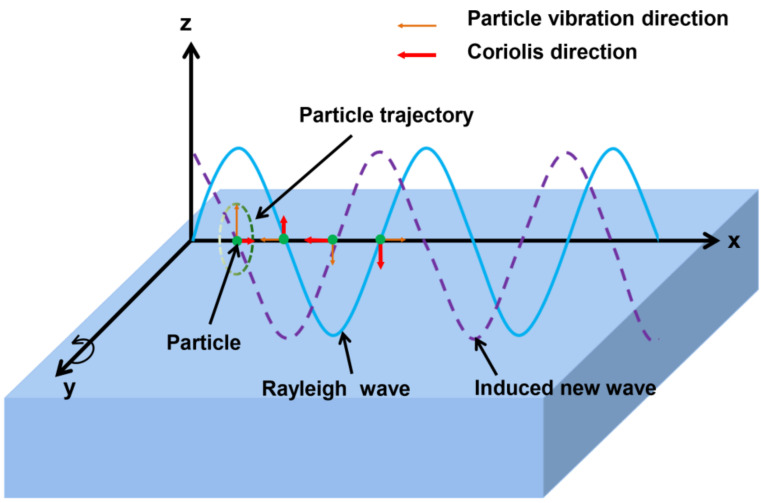
Schematic of the gyroscopic effect on the Rayleigh wave.

A coordinate system is established on the surface of a semi-infinite elastic substrate, as shown in Figure 2. The Rayleigh wave propagates along the x-axis, and its amplitude decays exponentially along the z direction. The particle moves in the plane composed of the x-axis and the z-axis. Since the amplitude of the SAW attenuates with the depth of the substrate, the vibration displacement of the particle is greatest on the surface of the substrate. To clearly demonstrate the SAW gyroscopic effect, we take a vibrating particle on the surface of the medium as an example. Due to the characteristics of Rayleigh waves, the motion trajectory of the surface particle is actually a counterclockwise ellipse, and the motion direction is the normal direction of the ellipse. When the substrate rotates counterclockwise around the y-axis, the vibrating particles in the SAW are also affected by inertial forces such as the Coriolis force and centrifugal force. Both of these forces induce a new SAW with a phase shift from the original SAW. This new SAW is coupled with the initial Rayleigh wave propagating on the substrate surface, which causes the movement trajectory of the particle to change and the velocity of the SAW to shift [34]. This is known as the SAW gyroscopic effect.

By comparing the wave equations of the elastic medium before and after loading a rotation, the influence of the Coriolis force and other factors on the SAW phase velocity can be observed. When the medium is not rotating, the general wave equation of the elastic wave in the medium is:

(1) $$\rho \frac{\partial^{2}u_{i}}{\partial t^{2}}=c_{ijkl}\frac{\partial^{2}u_{k}}{\partial x_{j}\partial x_{l}}$$

where ρ and c_ijkl_ are the density and elastic coefficient of the medium, respectively, with i, j, k, l = 1, 2, 3. When the medium rotates at an angular velocity Ω relative to the fixed coordinate system, the wave equation becomes [35]:

(2) $$\rho \frac{\partial^{2}u_{i}}{\partial t^{2}}=c_{ijkl}\frac{\partial^{2}u_{k}}{\partial x_{j}\partial x_{l}}-2\rho \varepsilon_{ijk}\Omega_{j}\frac{\partial u_{k}}{\partial t}-\rho \left(\Omega_{i}\Omega_{j}u_{j}-\Omega_{j}^{2}u_{i}\right)$$

In the above formula, the second term on the right side of the equal sign represents the Coriolis force, which depends linearly on the normalized rotation angle Ω/ω (Ω is the rotation vector, and ω is the angular frequency of the SAW device). The third term is the centrifugal force, which has a linear relationship with the square of Ω/ω. For piezoelectric materials, the wave velocity after loading a rotation can be calculated by jointly solving the wave equation and the electromagnetic wave equation with boundary conditions.

### 2.2. Simulation Details

**Figure 3:**
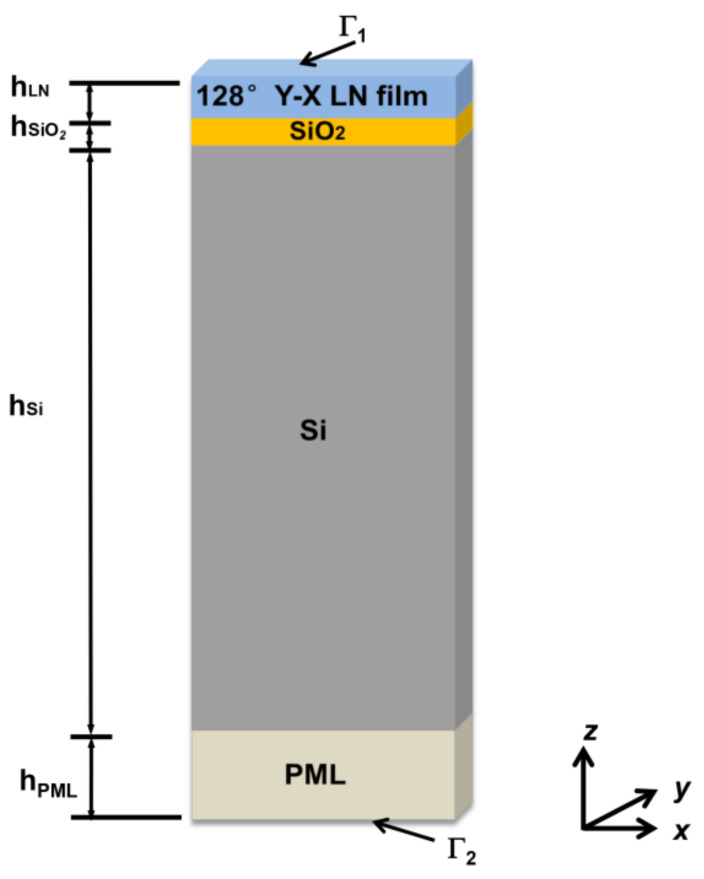
Three-dimensional (3D) periodic model of the LNOI substrate used in the finite element method (FEM).

The finite element method (FEM) is a common method for accurately simulating SAW devices, which is based on the piezoelectric constitutive equation and specific boundary conditions. The FEM is suitable for analyzing SAW characteristics in piezoelectric multilayer structures because it is highly flexible. The 3D model of the LNOI substrate was established in COMSOL, as shown in Figure 3. The model presents a periodic unit of the LNOI substrate. In this model, h_LN_ is the LN thin film thickness, and h_SiO2_ is the SiO_2_ layer thickness. The thickness of the Si substrate is replaced by a finite depth of 5 wavelengths (λ) and a perfectly matched layer (PML) at the bottom with a thickness of 1 λ [31]. The PML gradually absorbs mechanical and electrical interference by segments before they reach the boundary, resulting in a smaller model size [36]. In addition, the spurious resonance introduced by reflections at the top and bottom can be suppressed. The boundary conditions of the LNOI substrate model are as follows: (1) mechanical boundary conditions: the upper surface Γ_1_ is free, and the lower surface Γ_2_ is fixed; (2) electrical boundary conditions: the upper surface Γ_1_ has zero charge or is grounded, and the lower surface Γ_2_ has zero electric charge; (3) the front and back sides and the left and right sides have periodic boundary conditions; and (4) the LN/SiO_2_ interface and the SiO_2_/Si interface have continuous mechanical boundary conditions.

The vibration modes of the SAW in the LNOI substrate can be obtained by characteristic frequency analysis. Each propagation mode has a resonance frequency and anti-resonance frequency, which can be obtained from the vibration displacement distribution. If the electrode reflection effect is ignored, the two resonant frequencies are equal. Changing the electrical boundary condition of Γ_1_ to zero charge or grounded, the frequency of the vibration mode under the free and metalized boundary conditions can be obtained. The corresponding free and metalized SAW velocities (v_f_ and v_m_) can be calculated by multiplying the resonant frequency by the wavelength. K^2^ can be calculated by [37]:

(3) $$K^{2}=\frac{2(v_{f}-v_{m})}{v_{f}}$$

TCF is an important factor that characterizes the temperature stability of SAW devices and can be calculated by [23,24]:

(4) $$TCF=\frac{v_{35}-v_{15}}{20v_{25}}-\beta$$

where v_35_, v_25_ and v_15_ are the SAW phase velocities at 35 °C, 25 °C and 15 °C, respectively, and β is the coefficient of thermal expansion of the substrate.

The temperature dependence of the material constant is approximated by a second-order function and is expressed as follows [23]:

(5) $$X=X_{0}\left[1+\alpha_{1}\left(T-T_{0}\right)+\alpha_{2}{\left(T-T_{0}\right)}^{2}\right]$$

where X is the material constant at each temperature, X_0_ is the material constant at room temperature, T is the temperature, T_0_ is room temperature (25 °C in this paper), and α_1_ and α_2_ are the first- and second-order temperature coefficients of the material constants, respectively.

The gyroscopic effect can be simulated by loading a rotating frame with a given angular velocity into the LNOI model. For the LNOI model without rotation, the resonance and anti-resonance frequencies are equal. When the rotation angular velocity Ω is loaded, the resonant frequency splits into f_r+_ and f_r−_, where + and − represent the forward and reverse propagation directions of the SAW, respectively [38]. Hence, the SAW phase velocity v_r__±_ after loading a rotation is [14]:

(6) $$v_{r\pm }=f_{r\pm }\lambda$$

The SAW velocity shift caused by the gyroscopic effect can be calculated by:

(7) $$\Delta v_{\pm }=v_{r\pm }-v_{f}$$

Details of the material constants and temperature coefficients are listed in Table 1.

## 3. Results and Discussion

### 3.1. SAW Characteristics of the LNOI Substrate

**Figure 4:**
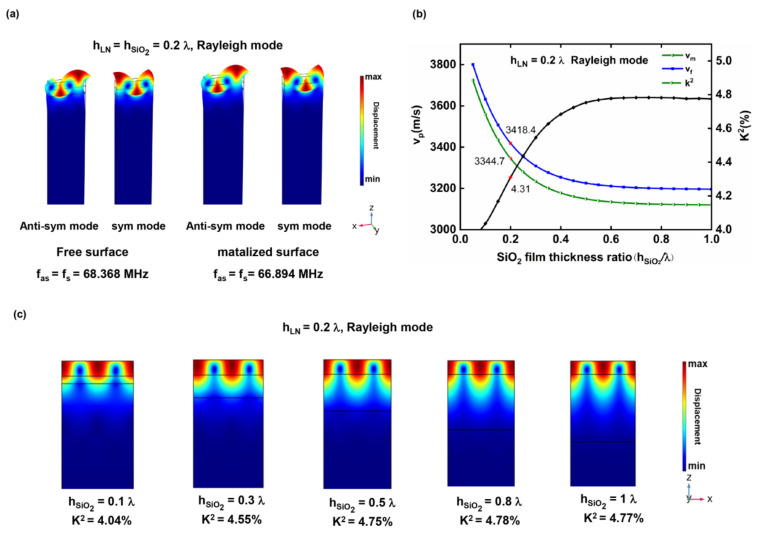
(**a**) Vibration mode of the LNOI substrate with h_LN_/λ = h_SiO2_/λ = 0.2; (**b**) v_f_, v_m_ and K^2^ dispersion curves of the LNOI substrate with h_LN_/λ = 0.2; (**c**) vibration mode of the LNOI substrate (h_LN_/λ = 0.2) with different h_SiO2_/λ.

There are multiple SAW modes in the LNOI substrate, such as Rayleigh and Love waves, which can be determined by the displacement distribution of the resonant modes. Rayleigh waves propagate on the surface of the medium, and their displacement components are mainly concentrated in the x and z directions, with almost no displacement components in the y direction. Figure 4a shows the anti-resonance and resonance modes of a Rayleigh wave on the LNOI substrate with an h_LN_ of 0.2 λ and an h_SiO2_ of 0.2 λ. The resonant frequencies are 68.368 MHz and 66.894 MHz under free and metalized surface conditions, respectively. Furthermore, the v_f_ and v_m_ values of this structure are calculated to be 3418.4 m/s and 3344.7 m/s, respectively, and K^2^ is 4.31%.

Generally, the SAW characteristic parameters of piezoelectric crystals are fixed. In contrast, the propagation characteristics of SAWs in piezoelectric multilayer structures are dispersive [39]. This means that the v_p_, K^2^ and TCF of the SAW are all related to the normalized thicknesses of the LN film (h_LN_/λ) and the SiO_2_ layer (h_SiO2_/λ). Figure 4b exhibits the variations in the v_f_, v_m_ and K^2^ of the Rayleigh wave with h_SiO2_/λ when h_LN_/λ is 0.2. v_f_ and v_m_ decrease sharply as h_SiO2_/λ increases from 0.05 to 0.5; v_f_ and v_m_ decrease slowly as h_SiO2_/λ increases further. In addition, when h_SiO2_/λ increases, the velocity difference between v_f_ and v_m_ gradually increases. When h_SiO2_/λ > 0.5, the difference between v_f_ and v_m_ gradually stabilizes. Therefore, the K^2^ of an LNOI substrate with an h_LN_/λ of 0.2 rises rapidly and then remains stable with increasing h_SiO2_/λ, as shown in Figure 4b. The variation trend of K^2^ can be illuminated more intuitively by comparing the vibration modes of LNOI substrates with different SiO_2_ layer thicknesses. Figure 4c shows the vibration mode of the LNOI substrate with an h_LN_/λ of 0.2 and an h_SiO2_/λ of 0.1, 0.3, 0.5, 0.8, and 1. Generally, the vibration of the Rayleigh wave is concentrated within a wavelength range under the surface of the medium. Therefore, the vibration of the Rayleigh wave in the LNOI substrate with an h_LN_/λ of 0.2 cannot be completely concentrated in the LN film. When h_SiO2_/λ is 0.1, the vibration is mainly concentrated in the LN film and SiO_2_ layer, and the Si substrate also has a partial vibration distribution. Since SiO_2_ and Si are not piezoelectric materials, the K^2^ of the LNOI substrate is only 4.04%, which is relatively small. When h_SiO2_/λ is increased to 0.3, the vibration is concentrated mostly in the LN film and SiO_2_ layer, with little leakage into the Si substrate. This is because the velocity of the Rayleigh wave in the SiO_2_ layer is smaller than that in the Si substrate and LN film, so the SiO_2_ layer functions similarly to a waveguide. Therefore, the K^2^ of the LNOI substrate increases to 4.55%. When h_SiO2_/λ is 0.5, the vibration is completely concentrated in the LN film and SiO_2_ layer, and K^2^ reaches 4.75%. As h_SiO2_/λ is increased to 0.8 and 1, the vibration distribution of Rayleigh waves is essentially the same as that when h_SiO2_/λ is 0.5 and K^2^ is 4.78% and 4.77%, respectively.

**Figure 5:**
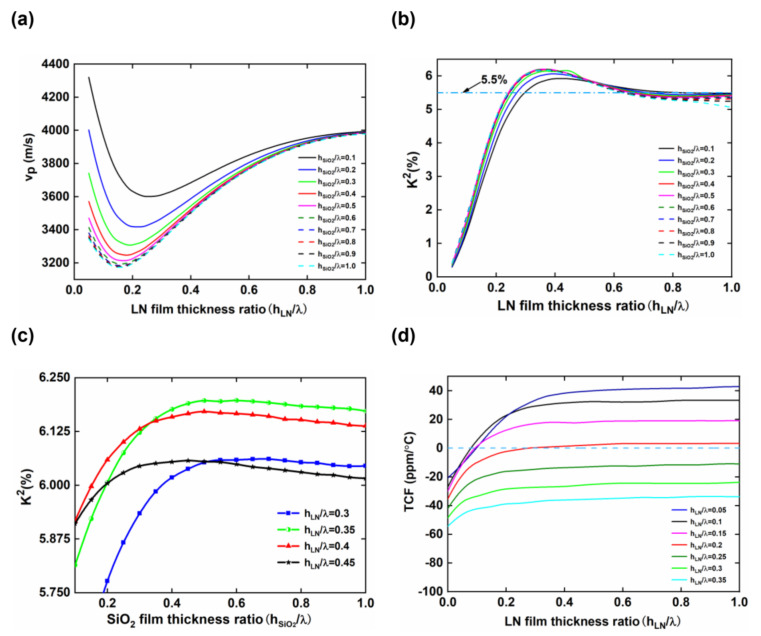
Variation in (**a**) v_p_ and (**b**) K^2^ with normalized thickness of the LN thin film (h_LN_/λ) in the LNOI substrate; (**c**) variation in K^2^ with normalized thickness of the SiO_2_ layer (h_SiO2_/λ) in the LNOI substrate when h_LN_/λ is between 0.3 and 0.45; (**d**) variation in TCF with normalized thickness of the SiO_2_ layer in the LNOI substrate when h_LN_/λ is between 0.05 and 0.35.

The center frequency of the SAW sensor is determined by the phase velocity. The variation trend of v_p_ versus h_LN_/λ on the free surface of an LNOI substrate with different h_SiO2_/λ is presented in Figure 5a. When h_SiO2_/λ is constant, the curves show that the v_p_ of the LNOI substrate rapidly decreases and then slowly increases with increasing h_LN_/λ. This is because the thickness of the LN film in the LNOI substrate directly affects the vibration distribution of Rayleigh waves. When h_LN_/λ is very small, most of the wave vibration in the LNOI substrate is concentrated in the SiO_2_ layer or Si substrate. At this time, the SiO_2_ layer only serves as an SAW propagation medium, so the v_p_ of the LNOI substrate drops rapidly. When h_LN_/λ increases to a certain value, more vibration energy is concentrated in the LN film, and the SiO_2_ layer acts as a waveguide layer, coupling more vibrations into the LN thin film and increasing the phase velocity. Finally, v_p_ approaches the LN crystal value of 3980 m/s. In addition, the change rates of the v_p_ of the LNOI substrate for different h_SiO2_/λ are often significantly different. Taking h_LN_/λ in the 0.05~0.15 range as an example, when h_SiO2_/λ is 0.1, v_p_ drops from 4320.9 m/s to 3739.05 m/s, and the velocity difference is as high as 581.85 m/s; when h_SiO2_/λ is 0.5, v_p_ decreases from 3471.3 m/s to 3216.05 m/s, and the velocity difference is only 255.25 m/s. This indicates that the v_p_ of an LNOI substrate with an h_SiO2_/λ of 0.1 is more sensitive to changes in the structural parameters.

K^2^, which is a parameter used to measure the conversion efficiency of piezoelectric materials between mechanical energy and electrical energy, affects the maximum design bandwidth and insertion loss of the device. A high K^2^ is an important criterion for high-performance SAW substrate materials. Figure 5b shows the dispersion curves of the calculated K^2^ of the LNOI substrate versus h_LN_/λ under different h_SiO2_/λ. The results demonstrate that the variation tendencies of the K^2^ curves are consistent when h_SiO2_/λ is a constant. The K^2^ of the LNOI substrate increases rapidly as h_LN_ increases and then declines slowly. Finally, the K^2^ of the LNOI substrate becomes approximately 5.3%, close to the value of the LN crystal [23]. The result indicates that the K^2^ of the LNOI substrate is mainly determined by the thickness of the piezoelectric layer LN. The blue shaded area in Figure 5b marks the area where K^2^ is greater than 5.5%. When h_LN_/λ is between 0.3 and 0.6 and h_SiO2_/λ is chosen as any value greater than 0.1, the K^2^ of the LNOI substrate is greater than 5.5%. To obtain the structural parameters of the LNOI substrate with a high K^2^, the value of K^2^ for different h_SiO2_/λ when h_LN_/λ is 0.3~45 is calculated. As shown in Figure 5c, although h_LN_/λ is different, h_SiO2_/λ has basically the same influence on the K^2^ of LNOI, showing a trend of an initial rapid rise and then maintaining stability. When h_LN_/λ is between 0.3 and 0.45 and h_SiO2_/λ > 0.4, the K^2^ of the LNOI substrate is greater than 6%. In particular, the K^2^ of the LNOI substrate reaches 6.2% when h_LN_/λ is 0.35 and h_SiO2_/λ is between 0.5 and 0.65.

The TCF reflects the stability of the central frequency of an SAW device as the temperature changes. The LN crystal has a large negative TCF of −75 ppm/°C, whereas SiO_2_ has a positive TCF. Therefore, a reasonable SiO_2_ thickness in the LNOI substrate can offset the influence of LN, producing a substrate with good temperature stability. Based on the characteristics of Rayleigh waves, when h_LN_ is larger, the energy of the SAW is basically concentrated in the LN film. The v_p_ and K^2^ of the LNOI substrate approach those of the LN crystal, and the TCF behaves similarly. Figure 5d shows the variation trend of the TCF with h_SiO2_/λ when h_LN_/λ is between 0.05 and 0.35. The SiO_2_ layer achieves temperature compensation for the negative TCF of the LNOI substrate with increasing h_SiO2_/λ. In particular, the TCF of the LNOI substrate increases from −18.75 ppm/°C to 3.19 ppm/°C when h_LN_/λ = 0.2, and the overall TCF is very small. When h_SiO2_/λ is 0.3, the TCF of the LNOI substrate is 0.3 ppm/°C, which is almost zero. In addition, the TCF of the LNOI structure is also close to zero for the following h_LN_/λ and h_SiO2_/λ parameter combinations: 0.05 and 0.097, 0.1 and 0.078, and 0.15 and 0.095, respectively.

### 3.2. Gyroscopic Effect of the LNOI Substrate

**Figure 6:**
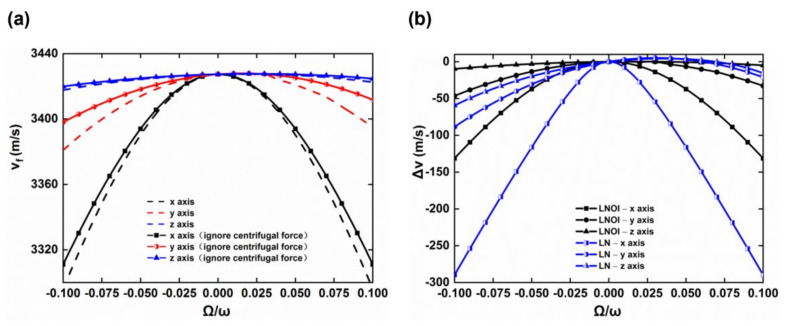
(**a**) Rayleigh wave phase velocity of the LNOI substrate (h_LN_/λ = 0.35, h_SiO2_/λ = 0.6) with rotation; (**b**) comparison of the phase velocity shift (Δv) depending on Ω/ω between the LNOI substrate (h_LN_/λ = 0.35, h_SiO2_/λ = 0.6) and LN crystal.

Based on the above research, the K^2^ of the LNOI substrate with h_LN_/λ = 0.35 and h_SiO2_/λ = 0.6 is as high as 6.2%, and the Rayleigh wave velocity is 3427.35 m/s. This LNOI substrate was selected to further study the gyroscopic effect after loading a rotation. Figure 6a illustrates the velocity curves of this LNOI substrate after rotation about the x-, y- and z-axes. The ordinate represents the Rayleigh wave velocity after loading a rotation; the horizontal coordinate represents the normalized rotation angular rate Ω/ω, where + and − correspond to clockwise or counterclockwise rotations of the LNOI substrate about an axis; and the dotted and solid lines indicate whether the influence of the centrifugal force is considered. As seen from the figure, the velocity changes of Rayleigh waves propagating along the x-axis of the LNOI substrate differ after rotation about the x-, y- and z-axes. When rotating about the x-axis, the SAW velocities after clockwise and counterclockwise rotations are the same, so the velocity change after loading a rotation cannot be detected by the differential scheme. When rotating about the y- and z-axes, the SAW velocities after clockwise and counterclockwise rotations are different, so the velocity change can be detected by the differential scheme. However, compared with rotation about the y-axis, the wave velocity changes after rotation about the z-axis are very small, rendering them more difficult to detect. From wave equation (2), the centrifugal force has a linear relationship with the square of Ω/ω. Figure 6a demonstrates that the influence of the centrifugal force becomes more obvious with increasing Ω/ω. The centrifugal force has a more noticeable effect on the Rayleigh wave velocity propagating along the x-axis when rotating about the y-axis. However, since the frequency of SAW devices is generally above MHz and the angular velocity measured daily is not very large, the influence of the centrifugal force was ignored in the early development of SAW gyroscopes. From Figure 6a, the effect of the centrifugal force on the SAW velocity is very small when ∣Ω/ω∣0.025 and can almost be ignored.

For SAW gyroscopes, the gyroscopic effect strength of the LNOI substrate is very important. Taking the LN crystal as the reference substrate, we contrast the change trends of the velocity shift (Δv) caused by the gyroscopic effect between the LNOI substrate (h_LN_/λ = 0.35, h_SiO2_/λ = 0.6) and the LN crystal, which is presented in Figure 6b. The results demonstrate that the Δv values of the LNOI substrate and the LN crystal have similar change trends after rotating about the x-, y-, and z-axes. Furthermore, the Δv of the LNOI substrate is smaller than that of the LN crystal, which means that the gyroscopic effect of the LNOI substrate is weaker than that of the LN wafer.

According to the literature [10,16], the gyro gain factor g can characterize the strength of the gyroscopic effect produced by a rotating medium, and the formula is:

(8) $$g=\frac{\Delta v/v_{f}}{\Omega /\omega }$$

g_+_ and g_−_, which represent propagation along the + and − directions of the rotation axis, can be calculated as:

(9) $$g_{+}=\frac{\Delta v_{+}/v_{f}}{\Omega /\omega }$$

(10) $$g_{-}=\frac{\Delta v_{-}/v_{f}}{\Omega /\omega }$$

Considering the advantages of a traveling wave SAW gyroscope with a differential scheme, a new gyroscope gain factor η is defined to characterize the strength of the gyroscopic effect in the differential scheme:

(11) $$\eta =g_{+}-g_{-}=\frac{\left(\Delta v_{+}-\Delta v_{-}\right)/v_{f}}{\Omega /\omega }=2\pi \frac{\left(f_{r+}-f_{r-}\right)}{\Omega }$$

The η of the LNOI and LN substrates can be calculated according to the above formula. The performance parameters of the two substrates are compared in Table 2. The results demonstrate that the K^2^ and TCF of the LNOI substrate (h_LN_/λ = 0.35, h_SiO2_/λ = 0.6) are better than those of the LN substrate, but its η values about the y-axis and z-axis are smaller than those of the LN substrate. Therefore, the structural parameters must be optimized to obtain a high-performance LNOI substrate.

**Figure 7:**
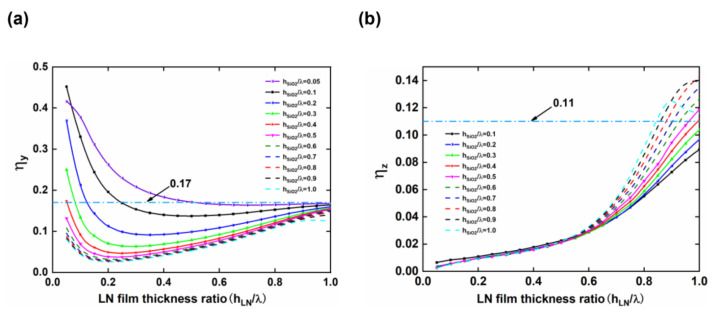
Variation in the gyroscope gain factors (**a**) η_y_ and (**b**) η_z_ with normalized thickness of the LN thin film in the LNOI substrate.

To compare the gyroscopic effect of LNOI substrates with different structural parameters, a large fixed rotational angular velocity of 3.0 × 10^7^ rad/s was applied to the LNOI substrate as it rotated about the y-axis and z-axis, and the η_y_ and η_z_ values were calculated for different structural parameters. Figure 7a shows the variation trend of η_y_ of an LNOI substrate with h_LN_/λ and h_SiO2_/λ after rotating about the y-axis. The blue dotted line indicates that the η_y_ of the LN crystal of 0.17. As shown in Figure 7a, the η_y_ of the LNOI substrate rapidly decreases and then slowly increases with increasing h_LN_/λ and finally approaches 0.17. The results indicate that the η_y_ of most LNOI substrates is less than 0.17. Only when h_LN_/λ is between 0.05 and 0.4 and h_SiO2_/λ is between 0.05 and 0.3 can an LNOI structure with an η_y_ greater than 0.17 be obtained. When h_LN_/λ is 0.05 and h_SiO2_/λ is 0.1, the η_y_ of the LNOI substrate can be as high as 0.45, which is much larger than that of the LN crystal. When h_SiO2_/λ is 0.05 and h_LN_/λ is any value, although the η_y_ of the LNOI substrate decreases from 0.42 to 0.16, the gyroscopic effect of the LNOI substrate is still stronger than or close to that of the LN crystal, which provides more space for h_LN_ parameter design. Figure 7b shows the variation trend of the gyroscopic effect of the LNOI substrate as it rotates about the z-axis as a function of the structural parameters, with the blue dotted line representing an η_z_ value of 0.11 for the LN crystal. The figure shows that when the LNOI substrate rotates about the z-axis, η_z_ increases with increasing h_LN_/λ. However, when h_LN_/λ is greater than 0.8 and h_SiO2_/λ is between 0.5 and 1.0, η_z_ is greater than 0.11. η_z_ has a maximum value of 0.143 when h_LN_/λ is 1.0 and h_SiO2_/λ is 0.85. By comparing Figure 7a,b, it can be concluded that the structural parameters for which the LNOI substrate has higher η_y_ and η_z_ are not in the same interval. Therefore, the structural parameters cannot be adjusted to ensure that the LNOI substrate has a strong gyroscopic effect when it rotates about the y-axis and z-axis at the same time. When designing the SAW gyroscope, the rotation axis can be selected according to the needs to design the structural parameters of the LNOI substrate.

To obtain high-performance SAW gyroscope substrate materials, Table 3 summarizes the structural parameters of LNOI substrates with optimal η_y_, η_z_, K^2^, and TCF are optimal values.

**Figure 8:**
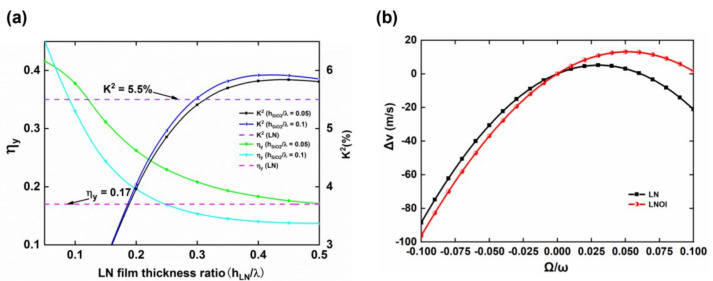
(**a**) Values of η_y_ and K^2^ for different structural parameters of the LNOI substrate and the LN wafer; (**b**) comparison of gyroscopic effects between the optimized LNOI substrate (h_LN_/λ = 0.2, h_SiO2_/λ = 0.05) and the LN wafer.

Table 3 shows that the structural parameters of the LNOI substrate are not similar when the four parameters are optimal. Therefore, the relationship between various performance parameters must be comprehensively considered according to the device requirements when designing LNOI substrates to obtain the optimal structural parameters. Taking a traveling wave SAW gyroscope with a differential structure as an example, more attention must be given to η_y_ and K^2^ since the differential structure can eliminate the influence of temperature. Figure 8a shows the variation curves of η_y_ and K^2^ when h_SiO2_/λ is 0.05 and 0.1, and h_LN_/λ is between 0.05 and 0.5. The dashed lines in Figure 8a indicate the η_y_ of the LN crystal of 0.17 and the K^2^ of 5.5%, respectively. Figure 8a demonstrates that the η_y_ and K^2^ of the LNOI substrate are greater than those of the LN crystal when h_SiO2_/λ is 0.05 and h_LN_/λ is between 0.3 and 0.5. However, the difference in η_y_ between the LNOI substrate and LN crystal is very small in this interval, and there is no significant improvement. Considering the parameters comprehensively, h_LN_/λ was chosen as 0.2 and h_SiO2_/λ as 0.05, and the η_y_ and K^2^ of this LNOI substrate were 0.26 and 3.96%, respectively. From Figure 8b, the gyroscopic effect of this LNOI substrate rotating about the y-axis is clearly better than that of the LN crystal. Additionally, the TCF of the structure is −18.75 ppm/°C, greatly improving the temperature stability. Therefore, this LNOI substrate can be used as a high-performance SAW gyroscope substrate material. When the wavelength is 50 μm, the thickness of the LN thin film is 10 μm and the thickness of the SiO_2_ layer is 2.5 μm. This LNOI substrate can be prepared by the micron-level LNOI substrate preparation scheme described above.

## 4. Conclusions

In this paper, the SAW propagation characteristics and the gyroscopic effect of an LNOI substrate were studied with 3D-FEM simulations. The variations in the v_p_, K^2^, TCF, η_y_ and η_z_ values of LNOI substrates with different structural parameters were obtained. The results indicate that the above parameters can be optimized by adjusting the thicknesses of the LN thin film and SiO_2_ layer. Furthermore, a SiO_2_ thin film with a positive TCF not only compensates for the negative TCF of LN but also enhances the K^2^ value of the LNOI substrate. When h_LN_/λ is 0.35 and h_SiO2_/λ is 0.6, the maximum K^2^ of the LNOI substrate is 6%. When h_LN_/λ is 0.2 and h_SiO2_/λ is 0.3, the TCF of the LNOI substrate is close to zero. When h_LN_/λ is 0.05 and h_SiO2_/λ is 0.1, the η_y_ of the LNOI substrate is 0.45. When h_LN_/λ is 1 and h_SiO2_/λ is 0.85, the η_z_ of the LNOI substrate is 0.143. Through the characteristic parameter trade-off, an LNOI substrate with an h_LN_/λ of 0.2 and an h_SiO2_/λ of 0.05 was obtained. Although the K^2^ of the LNOI substrate was 3.96%, the TCF was −18.75 ppm/°C and the η_y_ was 0.26. Compared with the 128°Y–X LiNbO_3_ crystal, the gyroscopic effect and temperature stability of this LNOI substrate were significantly improved. In addition, the LNOI substrate was easy to manufacture. The LNOI substrate can be used as a high-performance substrate material to improve performance and promote device miniaturization and integration of SAW gyroscopes.

## Figures and Tables

**Table 1:** Material constants and temperature coefficients used in the FEM simulation [23].

		Materials (T_0_ = 25 °C)			Temperature Coefficient α_1_ (10^−4^/°C)		
Material Constants		LiNbO_3_	SiO_2_	Si	LiNbO_3_	SiO_2_	Si
Elastic constant (10^11^ N/m^2^)							
C_11_		2.03	0.785	1.66	−1.74	2.39	0.68
C_12_		0.53	0.161	0.639	−2.52	5.84	−1
C_13_		0.75	0.161	0.639	−1.59	5.84	−1
C_14_		0.09	0	0	−2.14	0	0
C_33_		2.43	0.785	1.66	−1.53	2.39	−068
C_44_		0.6	0.312	0.796	−2.04	1.51	−0.44
C_66_		0.75	0.312	0.796	−1.43	1.51	−0.44
Piezoelectric constant (C/m^2^)							
e_31_		0.19			2.21		
e_15_		3.7			1.47		
e_33_		1.31			8.87		
e_22_		2.54			0.79		
Dielectric constant (10^−11^ F/m)							
ε_11_		38.9	3.2	10.36	3.23	0.263	0.68
ε_33_		25.7	3.2	10.36	6.27	−0.016	−1
Density (10^3^ kg/m^3^)							
ρ		4.7	2.2	2.33			
Thermal expansion coefficient (ppm/°C)							
β_1_		14.8	0.55	4.68			
β_3_		4.1	0.55	4.68			

**Table 2:** Characteristic parameters of the LNOI substrate and LN crystal.

Substrate	K^2^ (%)	TCF (ppm/°C)	v_p_ (m/s)	Rotation Axis	Ω/ω	Δv_+_ (m/s)	Δv_−_ (m/s)	η
128°Y–X LN	5.5	−75	3980	x	0.1	−289.75	289.75	0
				y	0.1	−21.5	−88.5	0.168
				z	0.1	−15.6	−59.4	0.11
LNOI (h_LN_/λ = 0.35, h_SiO2_/λ = 0.6)	6.2	−34.7	3427.35	x	0.1	−131.3	−131.3	0
				y	0.1	−32.65	−46.4	0.04
				z	0.1	−4.85	−9.7	0.014

**Table 3:** Structural parameter values of the LNOI substrate when η_y_, η_z_, K^2^ and TCF are optimal.

Substrate	v_p_ (m/s)	η_y_	η_z_	K^2^ (%)	TCF (ppm/°C)	h_LN_/λ	h_SiO2_/λ
1-LNOI	4320.9	0.45	0.0065	0.29	1.4	0.05	0.1
2-LNOI	3983.5	0.14	0.143	5.28	−84.5	1	0.85
3-LNOI	3427.45	0.04	0.014	6.2	−14.8	0.35	0.6
4-LNOI	3308.9	0.07	0.0094	4.55	0.31	0.2	0.3

## References

[1] Oh H., Lee K.J., Lee K., Yang S.S. (2015). Gyroscopes based on surface acoustic waves. Micro Nano Syst. Lett..

[2] Lee S.W., Rhim J.W., Park S.W., Yang S.S. (2007). A micro rate gyroscope based on the SAW gyroscopic effect. J. Micromech. Microeng..

[3] Zhang Y., Wang W. (2009). Enhanced Sensitivity of a Surface Acoustic Wave Gyroscope. Jpn. J. Appl. Phys..

[4] Wang W., Wang W., Liu J., Liu M., Yang S. (2011). Wireless and Passive Gyroscope based on Surface Acoustic Wave Gyroscopic Effect. Appl. Phys. Express.

[5] Fu C., Elmazria O., Sarry F., Mahalingam T., Yang S.S., Lee K. (2014). Development of wireless, batteryfree gyroscope based on one-port SAW delay line and double resonant antenna. Sens. Actuators A Phys..

[6] Kurosawa M., Fukuda Y., Takasaki M., Higuchi T. A surface acoustic wave gyro sensor. Proceedings of the International Solid State Sensors and Actuators Conference (Transducers '97).

[7] Varadan V., Suh W., Xavier P., Jose K., Varadan V. (2000). Design and development of a MEMS-IDT gyroscope. Smart Mater. Struct..

[8] Woods R.C., Kalami H., Johnson B. (2002). Evaluation of a novel surface acoustic wave gyroscope. IEEE Trans. Ultrason. Ferroelectr. Freq. Control.

[9] Oh H., Lee K., Yang S.S., Wang W. (2011). Enhanced sensitivity of a surface acoustic wave gyroscope using a progressive wave. J. Micromech. Microeng..

[10] Wang W., Liu J., Xie X., Liu M., He S. (2011). Development of a new surface acoustic wave based gyroscope on a X-112 degrees Y LiTaO3 substrate. Sensors.

[11] Wang W., Shao X., Liu X., Liu J., He S. (2014). Enhanced sensitivity of surface acoustic wave-based rate sensors incorporating metallic dot arrays. Sensors.

[12] Xu F., Wen W., Shao X., Liu X., Yong L. (2015). Optimization of Surface Acoustic Wave-Based Rate Sensors. Sensors.

[13] Lee M., Lee K. (2017). Enhancing the sensitivity of three-axis detectable surface acoustic wave gyroscope by using a floating thin piezoelectric membrane. Jpn. J. Appl. Phys..

[14] Sun X., Liu W., Shao X., Zhou S., Wang W., Lin D. (2018). Surface Acoustic Wave Gyroscopic Effect in an Interdigital Transducer. Sensors.

[15] Ge F., Zhao L., Zhang Y. (2021). Design and Optimization of a Novel SAW Gyroscope Structure Based on Amplitude Modulation with 1-D Phononic Crystals. Micromachines.

[16] Biryukov S.V., Schmidt H., Weihnacht M. Gyroscopic effect for SAW in common piezoelectric crystals. Proceedings of the 2009 IEEE International Ultrasonics Symposium.

[17] Wang W., Oh H., Lee K., Yoon S., Yang S. (2009). Enhanced Sensitivity of Novel Surface Acoustic Wave Microelectromechanical System-Interdigital Transducer Gyroscope. Jpn. J. Appl. Phys..

[18] Wang W., Xu F., He S., Li S., Lee K. (2010). A New Micro-rate Sensor Based on Shear Horizontal Surface Acoustic Wave Gyroscopic Effect. Jpn. J. Appl. Phys..

[19] Hu S.M., Hu Y.F., Cao X.S., Tian S.M. Gyro effect on surface acoustic wave propagation in a piezoelectric layered structure. Proceedings of the 14th Symposium on Piezoelectrcity, Acoustic Waves and Device Applications (SPAWDA).

[20] Zhou S., Lin D., Su Y., Zhang L., Liu W. (2020). Enhanced dielectric, ferroelectric, and optical properties in rare earth elements doped PMN-PT thin films. J. Adv. Ceram..

[21] Meng X., Zhang Z., Lin D., Liu W., Zhang L. (2021). Effects of particle size of dielectric fillers on the output performance of piezoelectric and triboelectric nanogenerators. J. Adv. Ceram..

[22] Cai C., Zhang D., Liu W., Wang J., Zhou S., Su Y., Sun X., Lin D. (2018). Synthesis, Giant Dielectric, and Pyroelectric Response of [001]-Oriented Pr^3+^ Doped Pb(Mg1/3Nb2/3)O_3_-PbTiO_3_ Ferroelectric Nano-Films Grown on Si Substrates. Materials.

[23] Tomar M., Gupta V., Mansingh A., Sreenivas K. (2001). Temperature stability of c-axis oriented LiNbO_3_/SiO_2_/Si thin film layered structures. J. Phys. D Appl. Phys..

[24] Tian X.-G., Liu H., Tao L.-Q., Yang Y., Jiang H., Ren T.-L. (2016). High-resolution, high-linearity temperature sensor using surface acoustic wave device based on LiNbO_3_/SiO_2_/Si substrate. Aip Adv..

[25] Ballandras S., Courjon E., Bernard F., Laroche T., Butaud E. New generation of SAW devices on advanced engineered substrates combining piezoelectric single crystals and Silicon. Proceedings of the Joint Conference of the IEEE International Frequency Control Symposium anEuropean Frequency and Time Forum (EFTF/IFC).

[26] Hsu T.-H., Su F.-C., Tseng K.-J., Li M.-H. Low loss and wideband surface acoustic wave devices in thin film lithium niobate on insulator (LNOI) platform. Proceedings of the IEEE 34th International Conference on Micro Electro Mechanical Systems (MEMS).

[27] Yan W., LIU X.-q., SHANG S.-l., Xun X. Fem Modeling Rayleigh Wave Sensors Based on GO/SiO_2_/128° YX-LiNbO_3_ Structures. Proceedings of the 2019 14th Symposium on Piezoelectrcity, Acoustic Waves and Device Applications (SPAWDA).

[28] Ma R., Liu W., Sun X., Zhou S. (2021). In-situ Process and simulation of high performance Piezoelectric-on-Silicon substrate for SAW sensor. Front. Mater..

[29] Poberaj G., Hu H., Sohler W., Günter P. (2012). Lithium niobate on insulator (LNOI) for micro-photonic devices. Laser Photonics Rev..

[30] Shuai Y., Gong C., Bai X., Wu C., Luo W., Böttger R., Zhou S., Tian B., Zhang W. (2018). Fabrication of Y128- and Y36-cut lithium niobate single-crystalline thin films by crystal-ion-slicing technique. Jpn. J. Appl. Phys..

[31] Hsu T.-H., Tseng K.-J., Li M.-H. (2020). Large Coupling Acoustic Wave Resonators Based on LiNbO3/SiO2/Si Functional Substrate. IEEE Electr. Device L.

[32] Lu R., Yang Y., Link S., Gong S. (2020). Low-Loss 5-GHz First-Order Antisymmetric Mode Acoustic Delay Lines in Thin-Film Lithium Niobate. IEEE T Microw. Theory.

[33] Tseng K.-J., Li M.-H. Frequency and coupling factor scaling of shear horizontal SAW resonators in LNOI platform. Proceedings of the 2020 Joint Conference of the IEEE International Frequency Control Symposium and International Symposium on Applications of Ferroelectrics (IFCS-ISAF).

[34] Zhou Y.H., Jiang Q. (2001). Efiects of Coriolis force and centrifugal force on acoustic waves propagating along the surface of a piezoelectric half-space. Z. Angew. Math. Phys..

[35] Yan Q., Wei Y., Shen M., Zhu J., Li Y. Theoretical and Experimental Study of Surface Acoustic Wave Gyroscopic Effect. Proceedings of the International Conference on Mechatronics & Automation.

[36] Sun X., Liu W., Ge S., Zhou S., Li X., Lin D. (2019). Achieving both high electromechanical response and stable temperature behavior in Si/SiO_2_/Al/LiTaO_3_ sandwich structure. AIP Adv..

[37] Luo J., Quan A., Chen F., Li H. (2017). Shear-horizontal surface acoustic wave characteristics of a (110) ZnO/SiO_2_/Si multilayer structure. J. Alloy. Compd..

[38] Sarapulov S.A., Kisilenko S.P. (1989). Gyroscopic effect in surface acoustic waves. Mekhanika Giroskopicheskikh Sist..

[39] Ro R., Lee R., Lin Z.-X., Sung C.-C., Chiang Y.-F., Wu S. (2013). Surface acoustic wave characteristics of a (100) ZnO/(100) AlN/diamond structure. Thin Solid Film..

